# Destructive influence of interlayer coupling on Heider balance in bilayer networks

**DOI:** 10.1038/s41598-017-15960-y

**Published:** 2017-11-22

**Authors:** Piotr J. Górski, Krzysztof Kułakowski, Przemysław Gawroński, Janusz A. Hołyst

**Affiliations:** 1 Faculty of Physics, Warsaw University of Technology, Koszykowa 75, 00-662 Warsaw, Poland; 2 AGH University of Science and Technology, Faculty of Physics and Applied Computer Science, al., Mickiewicza 30, 30-059 Kraków, Poland; 3 ITMO University, 49 Kronverkskiy av., 197101 Saint Petersburg, Russia

## Abstract

We consider the problem of Heider balance in a link multiplex, i.e. a special multiplex where coupling exists only between corresponding links. Numerical simulations and analytical calculations demonstrate that the presence of such interlayer connections hinders the emergence of the Heider balance. The effect is especially pronounced when the interactions between layers are negative, similarly as in antiferromagnetically coupled spin layers. The larger is the network, the narrower is the region of coupling parameters where the Heider balance can exist. If the interlayer couplings are of opposite signs and are strong enough, then the link dynamics can be reduced to the system of weakly coupled harmonic oscillators. For large strongly-coupled networks and randomly chosen initial conditions the probability of attaining the Heider balance decreases with the network size *N* as ${2}^{-{N}^{2}/2}$. Our finding can explain a lack of the Heider balance in many social systems, where multilayer structures mediate social interactions.

## Introduction

The concept of Heider balance (HB)^1^ is well-established in sociology^2^. Based on the Festinger theory of reduction of cognitive dissonance^3^, it has got a mathematical formulation^4^ in terms of networks. In a nutshell, the concept can be described as follows. In a fully connected social network, interpersonal relations can be friendly or hostile; hence links *x*_*ij*_ between pairs of nodes (*i*, *j*) are signed as ±1, respectively. The HB is attained if the relations fulfil the following axioms^5^:

- a friend of my friend is my friend,
- a friend of my enemy is my enemy,
- an enemy of my friend is my enemy,
- an enemy of my enemy is my friend.

Equivalently, the product of links in each triad $(i,j,k)$ is positive. As shown in^4^, the HB is equivalent to a partition of the network into two mutually hostile groups: each link between nodes from different groups is negative, and each link between nodes from the same group is positive. A special case is possible, where there is only one group and all links are positive. A natural context for applications of the idea of the HB is conflict; hence, the HB was quoted when referred to wars^6,7^.

Our aim here is to apply the concept of the HB to multilayer networks, where nodes are connected with multiple types of relations. These networks have got already a vast literature; for reviews see^8,9^. In graph theory, a multilayer concept is a very broad term. It encompasses networks with interconnected layers possibly of different sets of nodes and links, that are in general time-varying^10–17^. Such generalization leads to a complicated framework of network analysis. However, this approach is applied more and more often, because in many systems the division into layers brings a more natural description of reality and a more thorough insight into the system properties, structure and behaviour. In social networks^13–15,18–21^, for instance, agents can belong to the family, professional and friends group, each of which can pose a separate layer. Up to our knowledge, the HB in multilayers has not been discussed yet. However, the structure of triadic relations in multilayer models of society have been studied in^22–24^. For instance, it has been suggested^23^ that closing triads of positive links is one of the most essential principles in formation of such networks.

Our approach here is to investigate an influence of a coupling between two layers on the HB. The layers form a so-called *bilayer*, where all nodes exist in both layers and interlayer links connect only the same link replicas. The base parameter of the studied system is the strength of couplings between network layers, or more precisely relative strengths as compared to the influence of intralayer interactions. The problem of the strength of the interlayer coupling has already been analysed^21,25–34^ for other types of dynamics. Weakly coupled layers behave like separate networks, whereas the behaviour of layers, that are strongly connected, is significantly different from corresponding aggregated or single-layered networks. Moreover, when the coupling strength is increased, new phenomena, like phase transitions^21,25–27^, may be observed. Usually the symmetric coupling (that is the same for all layers) is assumed. Such an approach corresponds also to the study of magnetic layer systems where ferro- or antiferromagnetic interactions exist between spins belonging to different layers^35^. However, detailed analysis of real social systems^36,37^ might reveal that certain layers are much more important and, therefore, they are more influential. Only in few papers^25,32^ varying layer strengths were considered. Here, we do not limit the analysis and we study systems with asymmetric coupling coefficients modelling different mutual importance of network layers.

Within the common interpretation of the concept of HB, the weights of links are understood as relations between humans, friendly or hostile. The idea of multilayers introduced here means that there are two aspects of these relations, which interact and influence each other. As an example, consider two competing football teams formed of players from two city districts (or two nations); in each team there are players from both districts (or nations). Suppose that the links within the first layer are determined by the team membership, and the links within the second–by place of residence (or nationality). As long as the interlayer coupling constants are small, the HB can be attained separately in each layer; still the game is possible. If the relations are dominated by the membership, the game goes in a good sportsmanship. However, if the relations are dominated by the nationality, the only solution is to dissolve the teams, because chauvinistic prejudices are not a good base for football.

As another example consider a small family: parents and a child. In one layer, they are connected by links: wife-husband, mother-child, father-child. Suppose however, that the father teaches in a school where the child is a pupil, and the mother is a director in this school, or plays another important role. The school relations between the three, which form the second layer, can be different from the relations within the family. Yet, we can expect that the relation ‘father-child’ will be influenced by successes of the child in education, or by lack of them. A similar influence may appear for the other two relations. We note that the sign of the interlayer coupling is not necessarily positive. For example, the father wants to treat his child as any other pupil in class, and –exaggerating–treats her at school the more severely, the more affectionate is their relation at home.

Attention we pay to the case of one triad is justified in the case when the relation between three persons are so strong that they remain practically independent on their relations with people around them. The same assumption can be made on a social group of any size, and this is the way how we interpret the system size *N* in the text below.

In this paper the system dynamics is described by a set of differential equations, as in^38,39^. This involves both the intralayer connections and the interlayer coupling. Such a representation allows for analytical solution for small systems. In the absence of the coupling, the HB is known to be a generic solution^38–40^. We show, in particular, that in a system of coupled layers the HB is not generic and other scenarios are possible, e.g. one layer may take control over the other leading to the lack of the HB, or intralink oscillations may appear in the system. In the next section, the set of differential equations describing the system dynamics is given. In Results the first subsection is devoted to analytical solutions of some special cases. In particular, a stability analysis of system’s fixed points is performed for one triad; this approach can be treated as analogous to the calculation of the first term in a series expansion^41^. Further subsection provides numerical solutions of system dynamics. The probability of the HB is evaluated for different values of the interlayer coupling, by averaging over sets of different initial conditions. In the last section, the analytical and numerical results are discussed, and an attempt is made to broaden their interpretation.

## Model and Methods

### Network dynamics

In general, a multiplex $\overrightarrow{G}=({G}^{1},{G}^{2},\ldots ,{G}^{M})$ is a network of *N* nodes and *M* layers labelled by indices ($n\,=\,\mathrm{1,}\,\mathrm{2,}\ldots ,N$) and ($\alpha \,=\,\mathrm{1,}\,\mathrm{2,}\ldots ,M$), respectively. *G* ^*α*^ represents the network structure in the *α* layer. In each layer the same set of nodes is present, i.e. all the nodes have their replicas in all the layers. Nodes can be connected to different nodes only in the same layer, i.e. there are no interlayer links connecting different nodes. Interlayer connections exist between all the copies of the same node through all the layers.

**Figure 1:**
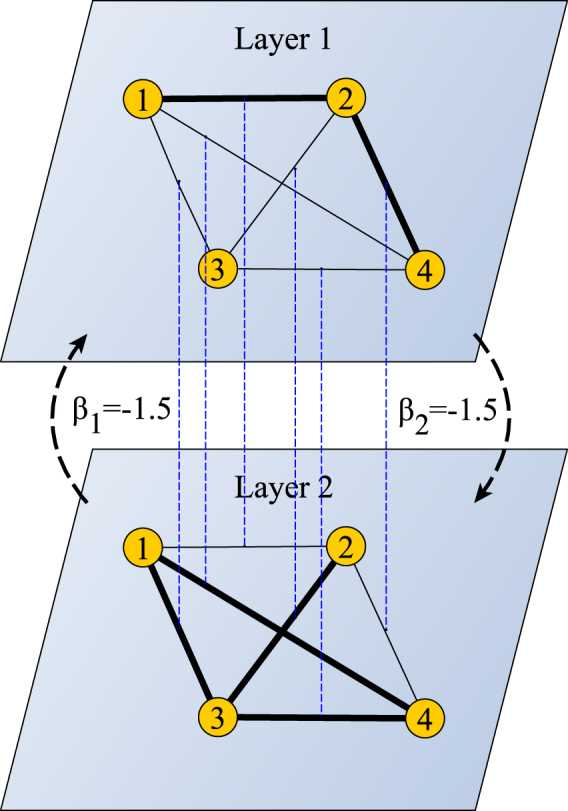
Example of link multiplex used in this paper. Interlayer connections are only between all the copies of corresponding links. The strength of influence of one layer on the other is determined by coupling coefficients (*β*_1_, *β*_2_). Here, intralayer connections represent friendly (+1) or hostile (−1) relations of type *α* between two nodes. Links +1/−1 are depicted by thick/thin lines. Intralayer interactions follow the dynamics that alone would lead to the HB. A strong negative coupling between both layers induces opposite values at corresponding links (see Results) and as a result no HB exists in the whole system.

Here, we modify a multiplex described above and we introduce a *link multiplex*, that is a multilayer network where the interlayer links exist only between all the copies of the same link through all the layers (Fig. 1). (A link multiplex can be transformed to a classic multiplex using a line graph transformation^42^). Let us consider a weighted^17,18^ bilayer network (i.e. link multiplex with two layers, *M* = 2) $\overrightarrow{G}=({G}^{1},{G}^{2})$. Each layer *G* ^*α*^ is represented by a time-dependent weighted adjacency matrix ${X}^{\alpha }(t)=\{{x}_{ij}^{\alpha }(t)\}$ (1).

1 $${\rm{\forall }}i,j\in \{1,2,\ldots ,N\}\,{\rm{\forall }}\alpha \in \{1,2\}:\{\begin{array}{ccc}{x}_{ij}^{\alpha } & \in  & [-1,1]\\ {x}_{ij}^{\alpha } & = & {x}_{ji}^{\alpha }\\ {x}_{ii}^{\alpha } & \equiv  & 0\end{array}.$$

Therefore, we assume a complete, undirected network with no self-loops (${x}_{ii}^{\alpha }\equiv 0$). An intralayer link is represented by a weight ${x}_{ij}^{\alpha }$, which signifies a type and a strength of connection between nodes *i* and *j* in the layer *α*. Basically, a variable ${x}_{ij}^{\alpha }$ means the relation (positive or negative, i.e. friendly or hostile) between agents *i* and *j* interacting in the *α* layer due to the *α*-type connections. Another interpretation is mentioned at the end of the paper.

When the interlayer coupling is taken into account, the time evolution of a weight ${x}_{ij}^{\alpha }$ is governed in our model by the equation:

2 $${\dot{x}}_{ij}^{\alpha }={(1-({x}_{ij}^{\alpha }))}^{2}(\frac{1}{N-2}\sum _{k\mathrm{=1}}^{k=N}{x}_{ik}^{\alpha }{x}_{kj}^{\alpha }+{\beta }_{\alpha }{x}_{ij}^{\alpha ^{\prime} }),$$

where *i* ≠ *j*, $\alpha ,\alpha ^{\prime} \in \mathrm{\{1,}\,\mathrm{2\}}$ and $\alpha \ne \alpha ^{\prime}$. Here a dot means the time derivative. *β*_*α*_ and ${\beta }_{\alpha ^{\prime} }$ are interlayer coupling constants: *β*_1_ is a measure of an influence of *links* of the second layer on *links* of the first layer, and *β*_2_ is a measure of an influence of *links* of the first layer on *links* of the second one. If *β*_1_ ≠ *β*_2_, the couplings are asymmetric. The sums are performed effectively over *N*−2 elements, as the diagonal elements ${x}_{ii}^{\alpha }$ of the matrices of weights are kept zero. Basically, the HB can be attained independently in both layers–so is in the case when ${\beta }_{1}={\beta }_{2}\,=\,0$ ^38^. Yet, as is demonstrated below, the coupling influences the state of the layers. In particular, if the coupling is negative and strong enough, the HB in one of the layers precludes it in the other layer.

The rationale for (2) is as follows. The prefactor $\mathrm{(1}-{({x}_{ij}^{\alpha })}^{2})$ is used in (2) to facilitate analytical discussion of the stability of fixed points^38^; it is more convenient than the alternative Heaviside function $H\mathrm{(1}-|{x}_{ij}^{\alpha }|)$. For each link ${x}_{ij}^{\alpha }$ the sum on the right-hand side (r.h.s.) is over all nodes *k* different from *i* and *j*. Recall that the network is fully connected, then each such node is a neighbour of both *i* and *j*. A positive sign of the product ${x}_{ik}^{\alpha }{x}_{kj}^{\alpha }$ means that the node *k* gives a contribution to an increase of ${x}_{ij}^{\alpha }$ towards its positive values, i.e. towards the HB in the triad $(ijk)$. On the contrary, a negative sign of the product ${x}_{ik}^{\alpha }{x}_{kj}^{\alpha }$ means that the node *k* gives a contribution to a decrease of ${x}_{ij}^{\alpha }$ towards its negative values, i.e. again towards the HB. In both cases, in a single-layered system the final state of the triad $(ijk)$ is such that the product ${x}_{ik}^{\alpha }{x}_{kj}^{\alpha }{x}_{ji}^{\alpha }$ is positive. The second term on the r.h.s. of (2) is just the interlayer coupling, its influence being the main subject of our text.

Although we study a network with two layers, one can generalize (2) for the network with *M* layers. In such a case the system can be described by $M(M-\mathrm{1)}$ coupling constants, and (2) can be written as

3 $${\dot{x}}_{ij}^{\alpha }={(1-({x}_{ij}^{\alpha }))}^{2}(\frac{1}{N-2}\sum _{k\mathrm{=1}}^{k=N}{x}_{ik}^{\alpha }{x}_{kj}^{\alpha }+\sum _{\alpha ^{\prime} }^{M}{\beta }_{\alpha \alpha ^{\prime} }{x}_{ij}^{\alpha ^{\prime} }),$$

where ${\beta }_{\alpha \alpha ^{\prime} }$ reflects an influence of the layer $\alpha ^{\prime}$ on the layer *α*, $i\ne j$, $\alpha ,\alpha ^{\prime} \in \mathrm{\{1,}\ldots ,M\}$, $\alpha \ne \alpha ^{\prime}$ and ${\beta }_{\alpha \alpha ^{\prime} }$ can be different from ${\beta }_{\alpha ^{\prime} \alpha }$.

Coming back to the case of two layers, let us look more closely at the coupling coefficients. Each link ${x}_{ij}^{\alpha }$ is directly influenced by $\mathrm{2(}N-\mathrm{2)}$ other neighbour connections from the same layer (*α*), and by a single link ${x}_{ij}^{\alpha ^{\prime} }$ from the other layer $(\alpha ^{\prime} )$. Therefore, the coupling coefficient ${\beta }_{\alpha }$ models the relative strength of an influence of the layer $\alpha ^{\prime}$, compared to an interaction within the layer *α*. In this sense, $|{\beta }_{\alpha }|\, > \,1$ ($|{\beta }_{\alpha }|\, < \,1$) signifies the system where an influence of all connections from the same layer is smaller (higher) than of the connection with the other layer. Further on in this paper, by a weak, moderate, strong and very strong coupling, we mean $|{\beta }_{\alpha }|\ll 1$, $|{\beta }_{\alpha }|\lesssim 1$, $|{\beta }_{\alpha }| > 1$ and $|{\beta }_{\alpha }|\gg 1$, respectively. Let us mention that in the case when ${\beta }_{1}={\beta }_{2}\, > \,0$, interlayer interactions are similar to a ferromagnetic coupling between thin magnetic layers^35^, where the links play the role of spins. In a similar way ${\beta }_{1}={\beta }_{2}\, < \,0$ corresponds to an antiferromagnetic coupling. The case when ${\beta }_{1}\ne {\beta }_{2}$ does not possess a clear interpretation in the language of magnetic systems because there is no interaction-like symmetry.

### Simulations

The set of system equations (2) have been solved numerically for different values of model parameters (*N*, *β*_1_, *β*_2_). Details of performed calculations are given in Supplementary Methods. Simulations have been performed for an ensemble of initial conditions generated from the uniform distribution, and have been repeated 10^2^–10^5^ times depending on the coupling coefficients. The following quantities have been measured: (i) *P*_*HB*_–probability of the HB, (ii) *P*_*s*_–probability of achieving a fixed point, (iii) a mean value $\langle S\rangle$ of interlayer link order (ILO) between corresponding links in different layers, where *S* is defined as:

4 $$S=\frac{2}{N(N-\mathrm{1)}}\sum _{i > j}^{N}sgn({x}_{ij}^{1}{x}_{ij}^{2})\mathrm{.}$$

The mean 〈*S*〉 varies from −1 to 1. A system is in a ferro- or antiferromagnetic ILO when $\langle S\rangle  > 0$ or $\langle S\rangle  < 0$, respectively. A system with $\langle S\rangle =1$ is in a complete ferromagnetic order, and it consists of two identical layers (in respect of signs of the links). A system with $\langle S\rangle =-1$ is in a complete antiferromagnetic order, and consists of two opposite layers, i.e. layers where all pairs of corresponding links have different signs.

## Results

### Analytical approach

Two processes are driving the system dynamics: (A) the tendency towards the HB induced by intralayer interactions, and (B) the tendency towards the same or opposite values of corresponding links in different layers *α* and $\alpha ^{\prime}$. These processes are intertwined and their relative importance changes with the model parameters: the network size *N* and the coupling coefficients $({\beta }_{1},{\beta }_{2})$. Moreover, the system dynamics also depends on the random distribution, generating initial weights. The tendencies (A and B) are strictly related to the form of model equation (2). Each link is directly influenced by other links from the same layer and by its replica from the other layer. For a link ${x}_{ij}^{\alpha }$ the mean of neighbouring link products ($\overline{{{\rm{\Sigma }}}_{ij}^{\alpha }}\equiv \frac{1}{N-2}\sum {x}_{ik}^{\alpha }{x}_{kj}^{\alpha }$) drives this link towards a balanced state. We expect that in a random initial state this term is, with a large probability, close to zero. Yet in a balanced state for each positive link (+1) the mean $\,\overline{{{\rm{\Sigma }}}_{ij}^{\alpha }}=+1$, and for each negative link (−1) the mean $\overline{{{\rm{\Sigma }}}_{ij}^{\alpha }}=-1$. Moving away from $\,\overline{{{\rm{\Sigma }}}_{ij}^{\alpha }}\approx 0$ means that a system moves towards the HB. On the other hand, the influence of link’s replica (${\beta }_{\alpha }{x}_{ij}^{\alpha ^{\prime} }$) leads towards a system with a complete ferro- or antiferromagnetic ILO, i.e. the system where the signs of all the corresponding links in both layers are the same or opposite.

Considering these processes, one can classify ordered states of the system: (i) HB states, (ii) states with a complete ferromagnetic ILO of corresponding links (they can be HB or not) or (iii) states with a complete antiferromagnetic ILO of corresponding links (they cannot be HB). A complete ferromagnetic order does not preclude appearance of the HB in both layers. A balanced triad in one layer corresponds to the same balanced triad in the second layer. On the other hand, a complete antiferromagnetic order precludes appearance of the HB, because the change of signs of all links in a balanced triad destroys the balance.

Now, we shall present analytical solutions of system dynamics for some special cases.

#### Symmetric system

Let us start with a symmetric system, where both layers are coupled with the same strength (${\beta }_{1}={\beta }_{2}\equiv \beta$). If the coupling is weak ($|\beta |\ll 1$), the mutual influence of the layers is negligible and the HB may emerge in each of them independently. On the other hand, if the system exhibits a very strong coupling ($|\beta |\gg 1$), intralayer interactions in the system equations (2) are negligible, which leads to the solution ${x}_{ij}^{\alpha }\approx \pm {x}_{ij}^{\alpha ^{\prime} }$, where the sign (±) is determined by the sign of *β*. It also means, that (2) can be reduced to the form of equation of motion for an overdamped particle in a nonlinear potential $V({x}_{ij}^{\alpha })=\frac{1}{4}{\beta }_{\alpha }{({({x}_{ij}^{\alpha })}^{2}-1)}^{2}$ of Landau-Ginzburg theory of phase transitions^43^

5 $${\dot{x}}_{ij}^{\alpha }={\beta }_{\alpha }(1-{({x}_{ij}^{\alpha })}^{2}){x}_{ij}^{\alpha }\mathrm{.}$$

The stable solutions of this equation are ${x}_{ij}^{\alpha }=\pm 1$, depending on initial conditions. It means that a very strong coupling limit leads to random extremal values of weights $\{-\mathrm{1,}+\mathrm{1\}}$.

In the case of very strong ferromagnetic coupling ($\beta \gg 1$), after a transient period the bilayer network comprises two identical layers, thus the HB may appear in the system. However, for larger *N* it is very unlikely. In total, there are ${2}^{N(N-\mathrm{1)/2}}$ possible configurations of link signs. As the initial state is determined by initial conditions, we can assume that each configuration is equally probable. When the HB is observed, then the nodes can be separated into two “hostile” groups *A* and *B*. Inside these groups all links are positive (±1), while links connecting nodes from *A* and *B* are negative (−1). Adding one more node *d* to this network, the HB can be sustained in two ways: (1) by adding this node to group *A*, which means setting all the links connecting node *d* with the nodes from *A* to ±1 and all the links connecting node *d* with nodes from *B* to −1, or (2) by adding this node to group *B* and setting all the links inversely than described above. It means, that in the network of size *N* + 1 there is twice as many possible HB configurations, than in the network of size *N*. For *N* = 3 there are four configurations with the HB. As initial conditions are randomly chosen, the probability of achieving the HB is

6 $${P}_{HB}\mathop{=}\limits^{\beta \gg 1}\frac{{2}^{N-1}}{{2}^{N(N-\mathrm{1)/2}}}={2}^{-(N-\mathrm{1)(}N-\mathrm{2)/2}}\mathrm{.}$$

In the case of $\beta \ll -1$, the HB in the whole system is impossible. If the HB is observed in one of the layers, then taking any balanced triad and *inverting* the signs always creates an *unbalanced* triad. Therefore, the HB in one layer prevents the HB in the second layer. It means that in such a situation, the symmetry between layers is spontaneously broken in a specific way since, although both layers are equivalent one to another in the sense of equations of motion, one of the layers is balanced and the other is not. However, because of negligible intralayer interactions different links in the same layer are independent of each other, and as a result the emergence of the balanced state is only the chance event. Its probability (i.e. attaining the HB in one layer, ${P}_{\mathrm{1,}HB}$) decays with the network size as ${P}_{\mathrm{1,}HB}\,=\,2{P}_{HB}$, where ${P}_{HB}$ is given by (6). Let us note that for $N\,=\,3$: ${P}_{\mathrm{1,}HB}\,=\,1$, because in all 8 configurations one of the layers is balanced.

#### One triad

The simplest case of *N* = 3 can give an insight into the behaviour of the system. The coupling between links in different layers is kept as above. For one triad, (2) can be written as follows:

7 $$\begin{array}{rcl}\dot{x} & = & \mathrm{(1}-{x}^{2})(yz+{\beta }_{1}u)\\ \dot{y} & = & \mathrm{(1}-{y}^{2})(zx+{\beta }_{1}v)\\ \dot{z} & = & \mathrm{(1}-{z}^{2})(xy+{\beta }_{1}w)\\ \dot{u} & = & \mathrm{(1}-{u}^{2})(vw+{\beta }_{2}x)\\ \dot{v} & = & \mathrm{(1}-{v}^{2})(wu+{\beta }_{2}y)\\ \dot{w} & = & \mathrm{(1}-{w}^{2})(uv+{\beta }_{2}z)\end{array}$$

For the sake of simplicity we modified the notation, exclusively in this subsection. Then, the links in the first layer are $x,y,z$, and those in the second layer are $u,v,w$. In these equations, the variable *x* is coupled to the variable *u*, *y* to *v* and *z* to *w*.

It is easy to see that a stable fixed point of (7) can exist provided that all the weights are equal ±1. Simple but tedious analytical calculations indicate also, that there are no other stable fixed points; see Supplementary Note. Thus, let us consider the set of stable fixed points. For (7), the Jacobian is diagonal (see Supplementary Note), and the conditions of stability are:

8 $$\begin{array}{lll}x(yz+{\beta }_{1}u) &  >  & 0\\ y(zx+{\beta }_{1}v) &  >  & 0\\ z(xy+{\beta }_{1}w) &  >  & 0\\ u(vw+{\beta }_{2}x) &  >  & 0\\ v(wu+{\beta }_{2}y) &  >  & 0\\ w(uv+{\beta }_{2}z) &  >  & 0\end{array}$$

Note that $$x,y,z,u,v,w\in \{-\mathrm{1,}+\mathrm{1\}}$$.

**Figure 2:**
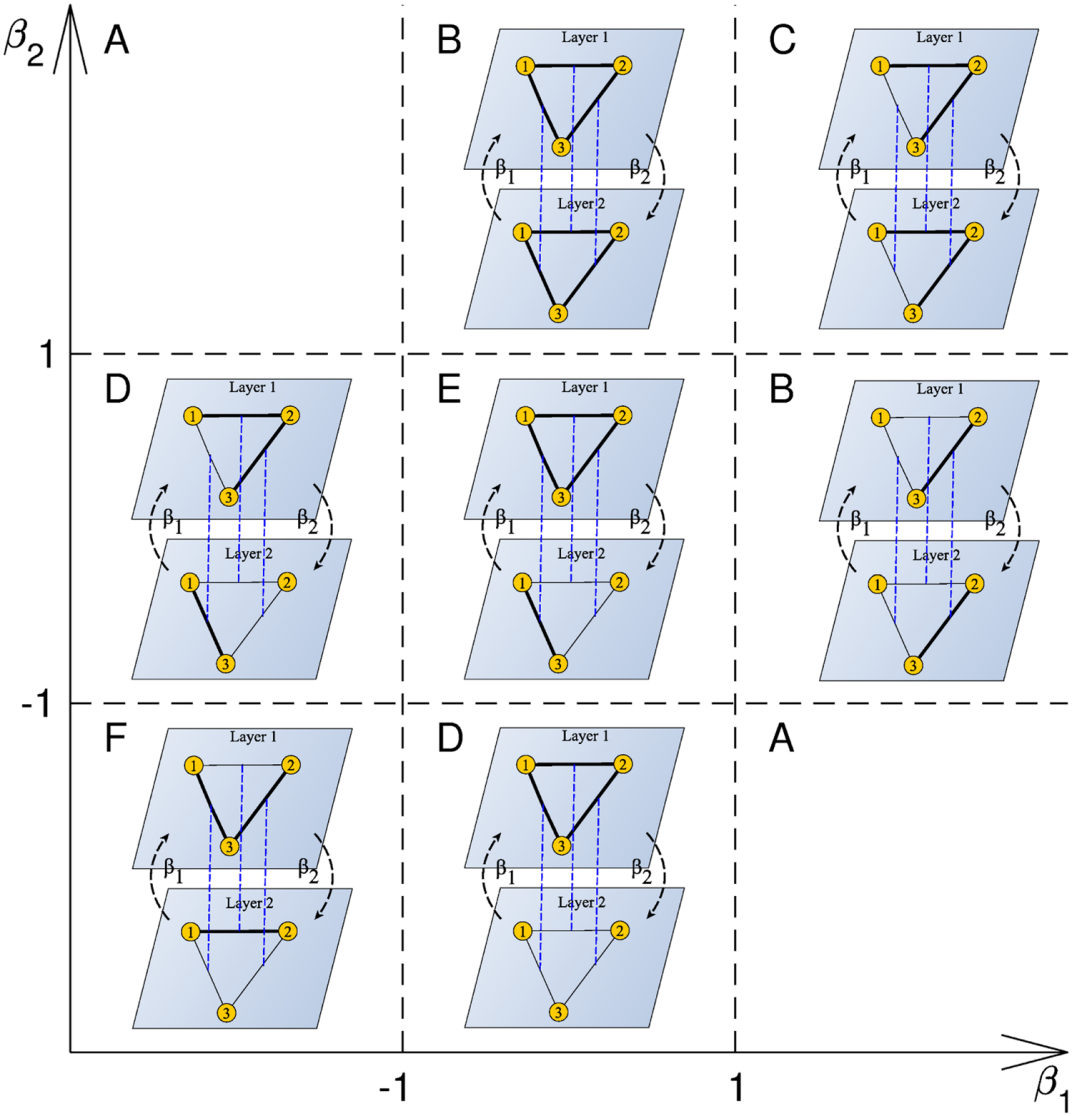
Examples of stable fixed points that a one-triad system may reach depending on the coupling coefficients. Fixed points with the HB have been chosen only if the HB is the only possible outcome. Links +1/−1 are depicted by thick/thin lines. Regions in the coordinate system have been named from **A** to **F**. Due to symmetry reasons some regions have the same names. No stable fixed point exists in **A**. The HB is the only possible outcome in **B** and **E**. The HB may also appear in **C**. Stable fixed points without the HB occur in **D** and **F**. Corresponding links in both layers are expected to be the same in **B** and **C** and opposite in **D** and **F**. See the discussion in text.

Depending on the values of coefficients on the plane with (${\beta }_{1},{\beta }_{2}$) as coordinates, we have identified 6 parameter regions leading to different system behaviour (Fig. 2). All the unique cases are listed below:

- Region E ($|{\beta }_{1}|\, < \,1$ and $|{\beta }_{2}|\, < \,1$): the solutions are stable as in the case of single layer iff $xyz=+1$ and $uvw=+1$, i.e. the HB is obtained.
- Region B (e.g. $|{\beta }_{1}| < 1$ and ${\beta }_{2} > 1$): the stability conditions impose the HB in the first layer, i.e. $xyz\,=\,1$, and they also impose the equality of weights in the second layer to those in the first layer. Therefore, the HB is restored again, and a complete ferromagnetic ILO is reached.
- Region C (${\beta }_{1}\, > \,1$ and ${\beta }_{2}\, > \,1$): the stability conditions are reduced to the equality of corresponding weights in both layers, whatever the signs of particular links are. Then, the presence of the HB depends on the initial values of weights. For example, if all weights are positive at time *t* = 0, the system is driven to the so-called paradise, where all weights are equal to ±1. This state is balanced in the sense of Heider. However, if all weights are initially negative and strong enough, the attained stable fixed point can be with all weights equal to −1, which is unbalanced in the sense of Heider. Summing up, if the initial weights’ distributions are symmetric around zero, we can expect the HB in about 50% of program runs (6).
- Region D (e.g. $|{\beta }_{1}|\, < \,1$ and ${\beta }_{2}\, < \,-1$): the stability conditions are $xyz\,=\,1$, $ux=-1$, $vy=-1$, and $wz=-1$. We have the HB in one layer and lack of the HB is extorted in the other. Therefore, the triad is unbalanced and links in both layers are of opposite signs.
- Region F (${\beta }_{1} < -1$ and ${\beta }_{2} < -1$): the stability conditions are that links in both layers are of opposite signs. If the HB is present in the first layer, it cannot be present in the other.
- Region A (e.g. ${\beta }_{1}\, > \,1$ and ${\beta }_{2} < -1$): the stability conditions cannot be fulfilled. In such a system for one of the layers the stability requires links equal to those in the other layer, whereas for the other layer the stability requires opposite links in both layers.

#### Strong coupling

Assuming that $|{\beta }_{1}|\approx |{\beta }_{2}|\gg 1$, ${\beta }_{1}{\beta }_{2}\, < \,0$, and that for each relation separately both replicas’ absolute values of weights ${x}_{ij}^{\alpha }$ are much smaller than 1, the equation of system dynamics for each link reduces to the harmonic oscillator equation:

9 $${\ddot{x}}_{ij}^{\alpha }-{\beta }_{1}{\beta }_{2}{x}_{ij}^{\alpha }=0$$

The detailed justification of this approximation is given in the Supplementary Note.

Oscillations are possible also for other ranges of the coefficients, e.g. for ${\beta }_{1}\, > \,1$ and $-1\, < \,{\beta }_{2}\, < \,0$. In such regions of coefficients two kinds of solutions are possible (a stable fixed point or periodic oscillations), and the result depends on initial conditions. This can be demonstrated for *N* = 3 and the limit case: ${\beta }_{1}\to \infty$. In this case, equation (7) for the variables $x,y,z$ allow to write $sgn(\dot{x})=sgn(u)$, etc. We expect that after a short time, $x=sgn(u)$. Then equation (7) for the variables $u,v,w$ can be written as $\dot{u}\,=\,\mathrm{(1}-{u}^{2})(vw+{\beta }_{2}sgn(u))$, etc. A sufficient condition for the oscillations is that $|vw| < -{\beta }_{2}$, and analogically $|wu| < -{\beta }_{2}$, $|uv| < -{\beta }_{2}$, because in this case either $u\, > \,0$ and $\dot{u}\, < \,0$, or $u\, < \,0$ and $\dot{u}\, > \,0$, and similarly for *v* and *w*. The volume in the space $u,v,w$ given by the conditions $|vw| < -{\beta }_{2}$, $|wu| < -{\beta }_{2}$, $|uv| < -{\beta }_{2}$ can be approximately evaluated as ${R}^{\mathrm{3/2}}$, where *R* is the area in the plane $(u,v)$ given by the condition $|uv| < -{\beta }_{2}$. In this way we get the probability of oscillations ${[-{\beta }_{2}\mathrm{(1}-ln(-{\beta }_{2}))]}^{\mathrm{3/2}}$ and, consequently the probability of reaching stationary point *P*_*s*_.

10 $${P}_{S}=1+{[{\beta }_{2}\mathrm{(1}-\mathrm{ln}(-{\beta }_{2}))]}^{\mathrm{3/2}}$$

This expression can be compared to the probability of non-stationary behaviour, as obtained numerically for large positive values of ${\beta }_{1}$ and $-1 < {\beta }_{2} < 0$. This comparison is shown below by numerical simulations in subsection Oscillations.

### Numerical results

Numerical simulations have been validated using the analysis from previous subsection. In the next two subsections, results for symmetric systems and a one-triad structure ($N=3$) are presented. Later, the study of the HB is extended for larger networks.

#### Symmetric systems

**Figure 3:**
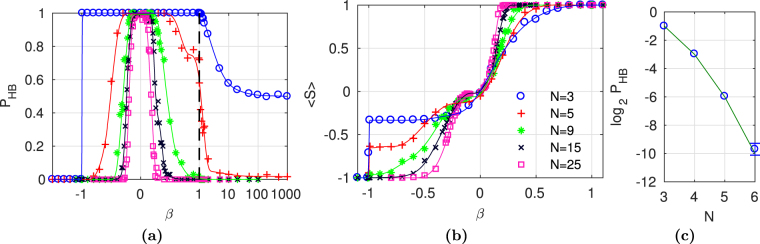
Mutual relationship of the probability ${P}_{HB}$ versus mean interlayer link order $\langle S\rangle$ for networks with symmetric coupling *β* for different network sizes *N*. Panels (a,b) show ${P}_{HB}(\beta )$ and $\langle S\rangle (\beta )$, respectively. The legend for (**a**,**b**) is given in panel (b). Fitted curves are only guide for eyes. In panel (a) horizontal axis is logarithmic for *β* > 1 and linear otherwise. Panel (c) shows decay of the probability *P*_*HB*_ as the function of the system size *N*. For large systems *P*_*HB*_ is infinitesimal. The figure shows the comparison between numerical (blue circle) and analytical (green line) results (6) for a system with very strong symmetric coupling between layers ($\beta \,=\,1000$). The error bar (standard deviation) in panel (c) is shown only when it is larger than the marker size. The calculations have been run at least 1000 times for each network in panels (a,b) and 10 000 times for each network in panel (c).

Let us consider a symmetric interlayer interaction (${\beta }_{1}={\beta }_{2}\equiv \beta$). Figure 3a shows the probability of the HB as a function of coefficient *β* for different network sizes. With the increasing coupling between layers, both negative and positive, the network is more and more unlikely to reach a balanced state. It appears that for a negative coupling $\beta  < -1$ the HB is never achieved, no matter the size of the network. The range of *β* leading to high ${P}_{HB}$ is decreasing with *N*. The HB is, to some extent, more probable for positive coupling coefficients. However, the difference between positive and negative couplings allowing the emergence of HB becomes marginal for larger *N*. The results for the simplest network ($N\,=\,3$) indicate, that the HB is always achieved when $|\beta |\, < \,1$. As expected for a very strong positive coupling, the probability of the HB decreases to 50%. This transition (from ${P}_{HB}\,=\,1$ to ${P}_{HB}\,=\,0.5$) is continuous. It is the opposite of the transition at $\beta =-1$, where the ${P}_{HB}$ changes discontinuously from 0 to 1.

Figure 3b presents the influence of coupling coefficients on ILO. Overall, for larger *N* the shape of $\langle S\rangle$ resembles the shape of tanh curve. For positive $\langle S\rangle$, there exists a critical *β* where a complete ferromagnetic ILO is reached. This critical value decreases with the network size. A complete antiferromagnetic ILO is achieved, regardless the network size for $\beta  < -1$. At $\beta =-1$ a discontinuous transition takes place. A change of $\langle S\rangle$ at the transition point decays quickly with growing *N*. When $|\beta |$ increases, the ordered state $\langle S\rangle =1$ is reached earlier, than the ordered state $\langle S\rangle =-1$. Remarkably, for $N\,=\,3$ when coupling coefficient is slightly larger than −1, the link order $\langle S\rangle$ is exactly $-\mathrm{1/3}$. Simultaneously, the HB is observed in the system, thus only 16 configurations are possible (+++, +−−, −+− and −−+ in each layer). One can easily check that identical layers give *S* = 1, whereas all the other configurations give $S=-\mathrm{1/3}$. There can’t be smaller ILO than −1/3 (because there is always the HB), but any higher result (achieved with same replica triads) would give a higher mean link order 〈*S*〉. Therefore, for such coupling only different HB states in layers can exist.

Looking at Fig. 3a and b one can notice, that the HB is possible provided there is no strong antiferromagnetic order (see also Supplementary Fig. S1). On the other hand, even the complete ferromagnetic ILO $\langle S\rangle =1$ can coexist with the HB. Notably, for the case of $N=3$ there are such values of *β*, that the probability ${P}_{HB}=1$ and the complete ferromagnetic ILO ($\langle S\rangle =1$) are observed simultaneously.

The plot in Fig. 3a makes evident, that the coupling value $\beta =1000$ is strong enough to observe a system where only interlayer relations are significant. That is why to verify the relation (6), the networks of size up to 6 nodes with symmetric coupling of $\beta =1000$ have been analysed (Fig. 3c). For larger networks the expected probability of the HB is very small, for instance statistically for *N* = 10 one needs to solve (2) for 2^36^ random initial conditions to achieve the HB once. The results presented in Fig. 3c confirm the theoretical estimation, given in (6). The HB is unlikely for *N* = 6 and it is almost impossible for networks *N* > 6.

#### One triad

Let us now consider the asymmetric case. It is a more natural network representation, as it models different importance of layers. The first step of analysis is to understand the one-triad system.

The coupling coefficients were taken from the set ${\bf{B}}=\{-\mathrm{1.5,}-\mathrm{1.4,}\ldots ,\,\mathrm{1.4,}\,\mathrm{1.5\}}\cup \{\pm \mathrm{0.99,}\pm \mathrm{1.01\}}$ $\cup \mathrm{\{2,}\,\mathrm{5,}\,\mathrm{10,}\,\mathrm{20,}\,\mathrm{50,}\,\mathrm{100,}\,\mathrm{200,}\,\mathrm{500,}\,\mathrm{1000\}}$, which results in 990 different pairs $({\beta }_{1},{\beta }_{2})\in {{\bf{B}}}^{2}$. Equations (7) have been solved for at least 100 (when any of the coefficients exceeded ${\beta }_{\alpha }\, > \,1.5$) or 1000 (otherwise) different random initial conditions.

**Figure 4:**
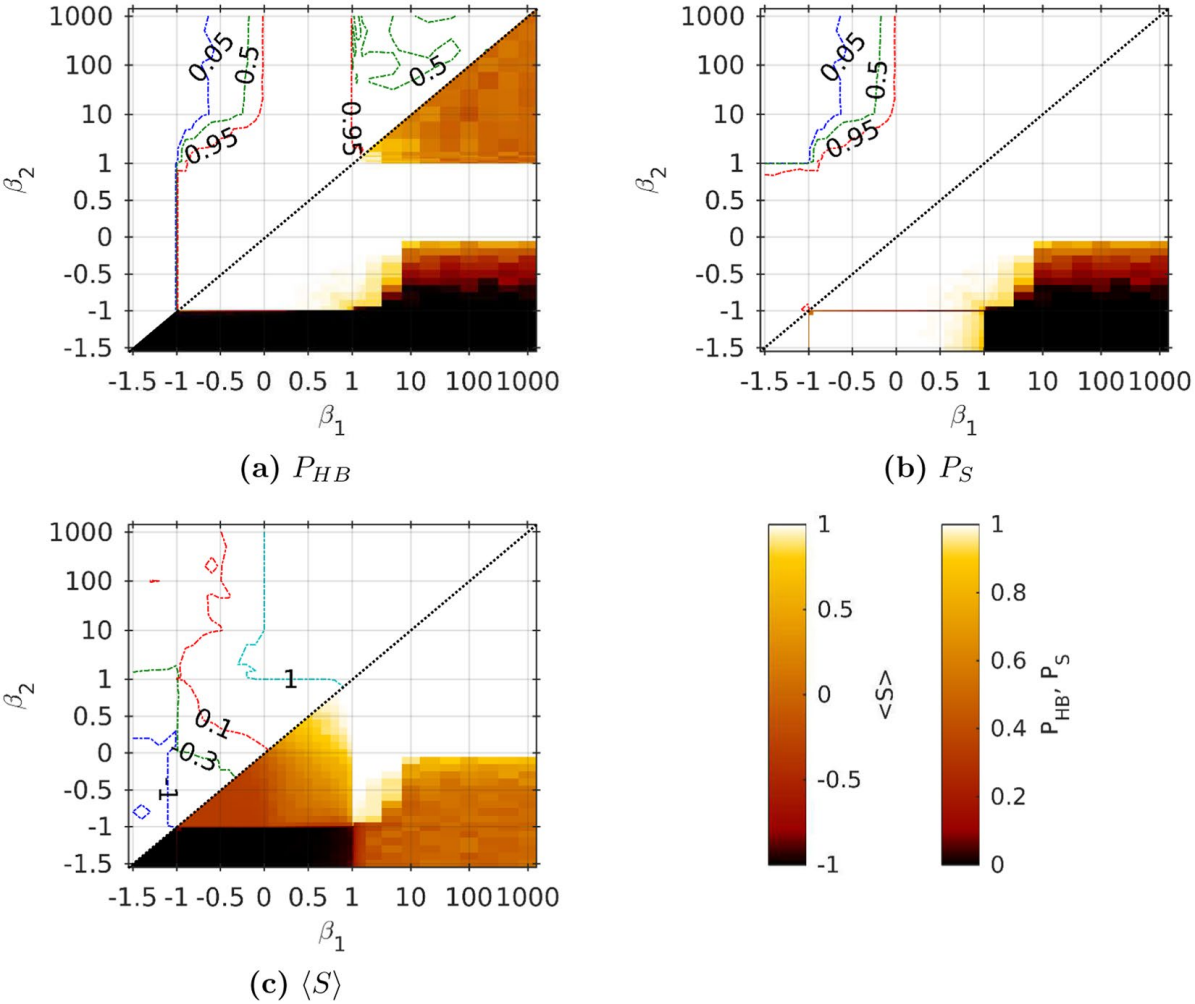
Results of simulations of one triad with various interlayer interactions. (**a**) Probability ${P}_{HB}$ of Heider balance, (**b**) probability *P*_*S*_ of a stationary solution of (2) and (**c**) mean interlayer link order $\langle S\rangle$ for different strengths of coupling for $N\,=\,3$. In each figure the upper triangle (${\beta }_{2}\ge {\beta }_{1}$) is a contour plot corresponding to a heat map in the lower triangle (${\beta }_{2}\le {\beta }_{1}$) excluding the data points on lines ${\beta }_{1}=\pm 1$ and ${\beta }_{2}=\pm 1$.

Figure 4 presents values of observables ${P}_{HB}$, ${P}_{S}$ and $\langle S\rangle$ for *N* = 3. Clearly, special cases of pairs $({\beta }_{1},{\beta }_{2})$ discussed in section with analytical solutions are easily distinguished. For $|{\beta }_{1}|\, < \,1$ and $|{\beta }_{2}|\, < \,1$, the system almost always reaches the HB (${P}_{HB}\approx 1$). Final weights ${x}_{ij}^{\alpha }$ are also stationary in other regions of the plane (${\beta }_{1},{\beta }_{2}$) (Fig. 4b), except two cases: (i) when any of the coupling values is equal or close to ±1 or (ii) when one coefficient is strong positive (>1) and the other is negative. A simple algebra leads to the conclusion that the former is the result of marginal stability; the eigenvalues of the Jacobian (8) are close to zero. The latter is related to a lack of a stationary solution; it will be discussed later.

Influence of the coupling coefficients on mean ILO is presented in Fig. 4c. High positive values of both coefficients lead to the network where both layers are the same, whereas completely opposite layers arise when ${\beta }_{\alpha } < -1$ and ${\beta }_{\alpha ^{\prime} }\, < \,1$. In this figure, the lack of stationarity manifests itself as $\langle S\rangle \ne \pm 1$ for large values of the coupling coefficients. Similarly as for symmetric coupling, in the region where both coefficients are slightly larger than −1, $\langle S\rangle =-\mathrm{1/3}$ and, thus, in each simulation a stationary fixed point containing nonidentical layers has been observed.

#### HB in larger networks

**Figure 5:**
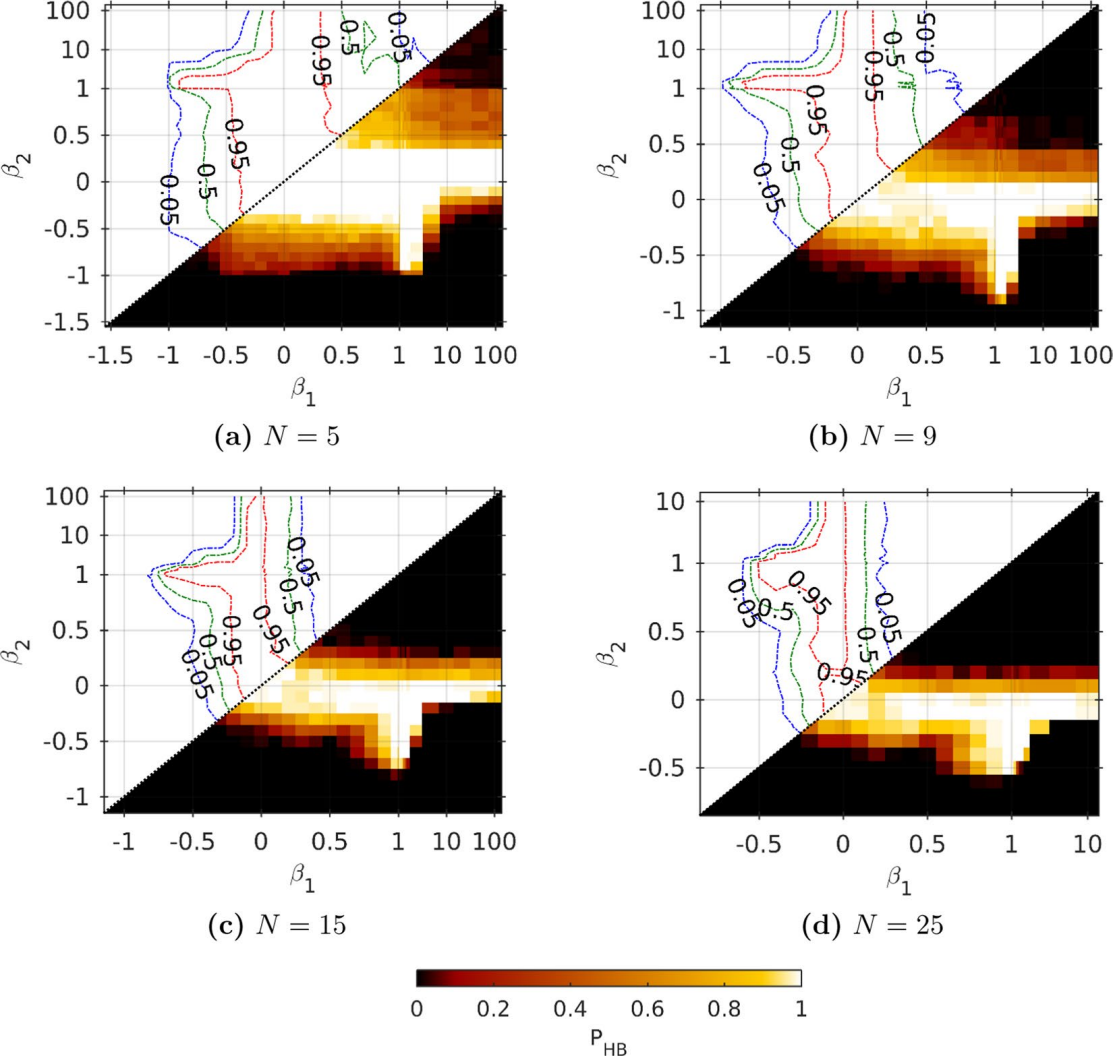
Probability ${P}_{HB}$ for different strengths of coupling for different network sizes. In each figure the upper triangle (${\beta }_{2}\ge {\beta }_{1}$) is a contour plot corresponding to a heat map in the lower triangle (${\beta }_{2}\le {\beta }_{1}$) excluding the data points on lines ${\beta }_{1}=\pm 1$ and ${\beta }_{2}=\pm 1$. The calculations have been run for each data point 100 times.

Figure 5 shows the probability of the HB in a bilayer of larger sizes ($N\,=\,\mathrm{5,\ 9,\ 15,\ 25}$). The coupling coefficients have been taken from the subsets of the previously mentioned set **B**, and for each pair of coefficients Equations (2) have been solved for at least 100 different random initial conditions.

Without the coupling [$({\beta }_{1},{\beta }_{2})\approx \mathrm{(0,}\,\mathrm{0)}$] the HB is always observed (${P}_{HB}\,=\,1$). Yet, in most cases the coupling between layers is disadvantageous for the HB. The larger is the network, the stronger is the effect. At Fig. 5 a few distinct regions can be distinguished, with boundaries dependent on the network size. There are regions with high HB probability: when layers are weakly or moderately coupled or when one of the layers is inducing the HB on the other. In the latter case, the other layer is strongly dependent and uninfluential. Regions with low *P*_*HB*_ emerge when both layers are strongly coupled or when one of the layers is inducing the lack of the HB on the other (again, the other layer is strongly dependent and uninfluential). In the first case, if $sgn({\beta }_{1})=sgn({\beta }_{2})$ then the system comprises two identical or two opposite layers, more precisely $S\approx sgn({\beta }_{1})$. Otherwise, an oscillating behaviour is observed.

As noted above, the boundaries of all the regions depend on the network size. With growing *N*, the boundaries are shifted closer towards the point $({\beta }_{1},{\beta }_{2})\,=\,\mathrm{(0,}\,\mathrm{0)}$. Regions with high *P*_*HB*_ become smaller. Transient areas between mentioned regions are also visible, and their sizes decrease with growing *N*.

#### Asymptotic solutions of system dynamics

In general, for given initial conditions the system may reach one of the ordered states (HB, *S* = 1 or *S* = −1). For other initial conditions the system may end up in a different order, may reach a fixed point not belonging to any ordered state, or may not reach any fixed point at all, ending up in link oscillations. However, for many coupling pairs (*β*_1_, *β*_2_) certain type of ordering is dominant, e.g. in all simulations the system ($N=5,{\beta }_{1}=0,{\beta }_{2}=0$) reached the HB, whereas the system ($N=5,{\beta }_{1}=100,{\beta }_{2}=100$) reached the complete ferromagnetic order. We have observed that for larger systems, the complete ferromagnetic order usually corresponds to ${P}_{HB}\approx 0$. The occurrence of a single type of solutions can be observed in diagrams with *β*’s as coordinates (Figs 4 and 5). As a result, for all network sizes we can identify distinct phases of coupling coefficients leading to the same solution type: (i) the non-stationary phase with oscillations (${P}_{S}\approx 0$) and two phases with stationary solutions– (ii) with ${P}_{HB}\approx 1$ and (iii) with ${P}_{HB}\approx 0$.

#### Oscillations

**Figure 6:**
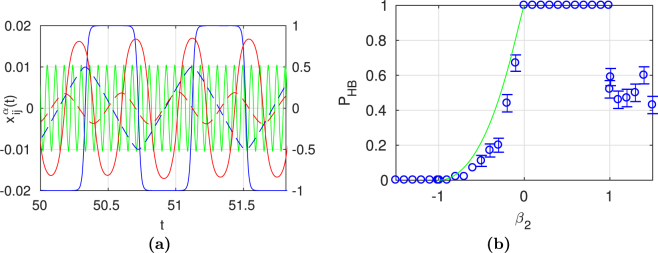
(**a**) Two examples of oscillating behaviour. Green curve (left axis) indicates harmonic oscillations of a selected link weight $x(t)$ in a system ($N\,=\,9$, ${\beta }_{1}\,=\,100$, ${\beta }_{2}=-100$). Blue and red curves (right axis) show trajectories forming anharmonic oscillations of two different pairs of corresponding links in a system ($N\,=\,9$, ${\beta }_{1}\,=\,100$, ${\beta }_{2}=-3$). Solid and dashed lines represent links from layer 1 and layer 2, respectively. (**b**) Destructive effect of interlayer coupling on HB. Analytical approximation (green line) follows (10): ${P}_{HB}=1-|{\beta }_{2}{|}^{\mathrm{3/2}}{\mathrm{(1}-ln|{\beta }_{2}|)}^{\mathrm{3/2}}$ for $N=3$, ${\beta }_{1}\to \infty$ and $-1 < {\beta }_{2} < 0$. Numerical simulations of a system ($N=\mathrm{3,}\,{\beta }_{1}=1000$) are presented as (blue circle). In the absence of HB the system exhibits oscillations for ${\beta }_{2}\mathrm{ < \; 0}$ or achieves unbalanced stationary solution for ${\beta }_{2} > 1$. Error bars (standard deviation) are shown only when they are larger than a marker size.

For the coupling coefficients of the same sign, the interaction between the layers may result in achieving a stationary state with a certain kind of ordering (HB or ILO). On the other hand, when ${\beta }_{\alpha }\, > \,0$ and ${\beta }_{\alpha ^{\prime} }\, < \,0$, both layers start to disturb each other, because the tendency towards (anti)ferromagnetic order is not consistent any more. The layer *α* tends towards $S\,=\,1$, and the layer $\alpha ^{\prime}$–towards $S=-1$. As a result, apart from stationary states, the outcome solution may be oscillating. More generally, oscillations are achievable when the signs of the coupling coefficients are opposite and at least one of the coefficients $|{\beta }_{\alpha }|$ is large enough. For networks up to $N\,=\,9$, the threshold value of the coupling ${\beta }_{s}$ above which oscillations are visible is close to 1; for larger *N*, it decreases slowly with the network size. When both coefficients are out of the range $(-{\beta }_{s},{\beta }_{s})$, oscillations seem to be the only solution. In general, oscillations can be anharmonic (see Fig. 6a). But as noticed in subsection with analytical calculations, in some circumstances we can also expect harmonic oscillations. Figure 6b gives a comparison between the analytical result (10) and numerical data of a system ($N=3,{\beta }_{1}=1000$). In the range $-1\le {\beta }_{2}\le 0$, the only possible solution types are the HB or oscillations, thus we have ${P}_{HB}\equiv {P}_{S}$. One can observe a good fit between numerical and analytical probabilities of stationary solutions. A small discrepancy between both results is because the approximate theoretical estimation (10) captures only the space of initial weights fulfilling a sufficient condition of the oscillations. Thus, the space of the necessary condition of achieving oscillations is larger than assumed.

## Discussion and Conclusions

Vast applications of multilayer models indicate, that multivalued links are useful in attempts to understand real systems. A model presented in this paper may describe cooperation in small groups, e.g. sport teams, work groups in companies, collaborating scientists. In these examples one layer is related to achieving a common goal, and another is related to personal prejudices, private opinions or willingness to communicate. In the introduction we described the case of two competing football teams, where the first layer corresponds to the team membership and the second–to nationality. Using the idea of coupling coefficients, a fair match is impossible if $|{\beta }_{2}|\, < \,1$ and ${\beta }_{1}\, > \,1$, that is if the relations are dominated by the nationality.

Our results allow to imagine yet another application of the HB, which relies on interpretation of network nodes as logical statements. Then, if two statements cannot be simultaneously true, their mutual relation is negative; otherwise is positive. As an example, suppose two economic strategies for a country. Each plan is internally consistent and perhaps even feasible, but each is designed according to entirely different vision. Each plan consists of a set of statements which form a network; the HB is possible there. Now, let us add the second layer - public opinion. It is not hard to imagine a polarization of political discourse such, that the vast majority of citizens qualifies each above statement as merely an expression of one of two opposite ideologies. To be precise, two statements are qualified as mutually consistent, if both can be reformulated to start from ‘the state should pay for…’ or both can be started from ‘the state should not pay for…’. Otherwise the statements are treated as mutually inconsistent; then in the second layer the relation between them is negative. A similar content-based classification has been applied in^44^. It is clear that the links in these two layers are of not necessarily of the same sign. Further, we have no arguments to deny the coupling between the layers, presumably asymmetric; in our terms, *β*_1_ ≠ *β*_2_. Then here again, the HB in the second layer can destroy the balance in the first, even if we would prefer the opposite. This conclusion is consistent with the role of simple ‘stories’ in politics, as has been convincingly discussed in^45^.

In conclusion, the introduced here concept of the link multiplex offers a new tool for investigations of the HB, that deals with an overall consistency of relations between system’s constituents. Numerical and analytical investigations show that the interlayer coupling tends to hinder the emergence of the HB. Since the dynamics in our model is driven by two different processes, diverse ordered states can emerge: Heider balance of links in a layer (due to intralayer interactions), and (anti)ferromagnetic order between corresponding links in both layers. Depending on the coupling coefficients, both kinds of ordering can be reached, only one of them is possible, or none of them occurs. The first two cases correspond to stationary states. In the third case the system may be also periodic in time.

Basically, the influence of the interlayer coupling depends on the values of the coupling constants. However, as there are two kinds of interactions (intralayer and interlayer), and only the first one promotes the Heider balance, the second interaction hinders it. For symmetric interlayer interactions (${\beta }_{1}={\beta }_{2}\equiv \beta$), where both layers are of equal importance, all solutions are stationary. In such a case the HB is likely to be achieved only when $|\beta |$ is small enough, i.e. the influence of the interlayer coupling is weaker than the influence of intralayer links. Positive and negative *β* are analogical to ferromagnetic and antiferromagnetic interactions, respectively. Ferromagnetic order of links from different layers can coexist with the HB over a larger range than antiferromagnetic order. When the coupling is very strong and positive ($\beta \gg 1$), then the probability of the HB decreases with the network size as ${2}^{-{N}^{2}\mathrm{/2}}$ (6). On the other hand, when the coupling is strong and negative ($\beta  < -1$), then the HB in the whole system is impossible. In the presence of asymmetric coupling, the obtained phase diagrams are more complex, as they contain balanced and unbalanced stationary phases as well as periodic oscillations.

We conclude that since the multilayer structure is a prevailing topology mediating social interactions, thus our finding can explain a lack of the Heider balance in many of such systems. Our model, however relatively simple, should find further extensions and applications to social structures, where conditions of consistence are meaningful.

### Data Availability

No datasets were generated or analysed during the current study.

## Electronic supplementary material

Supplementary Information
