# Supplementary material for: Destructive influence of interlayer coupling on Heider balance in bilayer networks
Source: Sci Rep. 2017 Nov 22;7:16047. doi: 10.1038/s41598-017-15960-y (PMC5700191; doi:10.1038/s41598-017-15960-y)
Supplement: Supplementary file 1 — Supplementary Information [file 41598_2017_15960_MOESM1_ESM.pdf]

Supplementary Information for: Destructive influence of interlayer coupling on Heider balance in bilayer networks

Piotr J Górski[1], Krzysztof Kułakowski[2], Przemysław Gawroński[2], and Janusz A Hołyst[1,3,*]

[1]Faculty of Physics, Warsaw University of Technology, Koszykowa 75, 00-662 Warsaw, Poland
[2]AGH University of Science and Technology, Faculty of Physics and Applied Computer Science, al. Mickiewicza 30, 30-059 Kraków, Poland
[3]ITMO University, 49 Kronverkskiy av., 197101 Saint Petersburg, Russia
[*]jholyst@if.pw.edu.pl

November 9, 2017

Supplementary Note

Fixed points in a one-triad network

The set of equations reads

$$\dot{x} = (1-x^2)(yz+\beta_1 u) \tag{1}$$
$$\dot{y} = (1-y^2)(zx+\beta_1 v) \tag{2}$$
$$\dot{z} = (1-z^2)(xy+\beta_1 w) \tag{3}$$
$$\dot{u} = (1-u^2)(vw+\beta_2 x) \tag{4}$$
$$\dot{v} = (1-v^2)(wu+\beta_2 y) \tag{5}$$
$$\dot{w} = (1-w^2)(uv+\beta_2 z) \tag{6}$$

The task is to find the fixed points and to determine their stability. The stability condition is that the real parts of all eigenvalues of the Jacobian at the fixed point are strictly negative.

The Jacobian is

$$\mathbf{J} = \begin{pmatrix} -2x(yz+\beta_1 u) & (1-x^2)z & (1-x^2)y & (1-x^2)\beta_1 & 0 & 0 \\ (1-y^2)z & -2y(zx+\beta_1 v) & (1-y^2)x & 0 & (1-y^2)\beta_1 & 0 \\ (1-z^2)y & (1-z^2)x & -2z(xy+\beta_1 w) & 0 & 0 & (1-z^2)\beta_1 \\ (1-u^2)\beta_2 & 0 & 0 & -2u(vw+\beta_2 x) & (1-u^2)w & (1-u^2)v \\ 0 & (1-v^2)\beta_2 & 0 & (1-v^2)w & -2v(wu+\beta_2 y) & (1-v^2)u \\ 0 & 0 & (1-w^2)\beta_2 & (1-w^2)v & (1-w^2)u & -2w(uv+\beta_2 z) \end{pmatrix}$$

In the simplest case $\beta_1 = \beta_2 = 0$ there is no coupling between the layers and the only stable points are when $xyz > 0$ and $uvw > 0$, what is just the condition of the Heider balance (HB). Also we have three lines of unstable fixed points $(x, 0, 0)$, $(0, y, 0)$ and $(0, 0, z)$, and the same for (u, v, w).

This is a special case of the more general situation, where $\beta_1 = 0$ and $\beta_2 \neq 0$. This is an example of the master-slave relation, as the layer (x, y, z) does not depend on the layer (u, v, w), but the opposite dependence persists. The Jacobian has a block-diagonal structure, with three eigenvalues equal to $-2xyz$; here again, the only stable solution for this layer is HB. For u, v, w we

get three equations of motion:

$$\dot{u} = (1-u^2)(vw+\beta_2 x) \tag{7}$$
$$\dot{v} = (1-v^2)(wu+\beta_2 y) \tag{8}$$
$$\dot{w} = (1-w^2)(uv+\beta_2 z) \tag{9}$$

Looking for stable fixed point for the whole system, we can distinguish some sets of equivalent configurations, and there is no need to consider each of them separately; then we can assume that either $x = y = z = +1$, or $x = y = -1$, $z = 1$. Let us consider the first case. Either some of the variables $u, v, w = \pm 1$, or the product of some pairs of them is equal to $-\beta_2$. Truly different options are as follows:

* $u, v, w = \pm 1$. Then the Jacobian is diagonal, and the eigenvalues are $-2u(vw+\beta_2), -2v(wu+\beta_2), -2w(vu+\beta_2)$. For $|\beta_2| < 1$ the condition of stability is HB again. For $\beta_2 > 1$, what is stable is the phase of 'paradise', i.e. $u = v = w = +1$. For $\beta_2 < -1$, the stable phase is $u = v = w = -1$, what is not HB.

* $u, v = \pm 1$ and $uv = -\beta_2$, what is not generic.

* $u = \pm 1$, $v = w = \mp\beta_2$; then $|\beta_2| < 1$. Then, two of eigenvalues are $\pm(1-\beta_2^2)$.

* $u = v = w = \pm\sqrt{-\beta_2}$. Then, $\beta_2 < 0$ and the related eigenvalues are $-e, -e, 2e$, where $e = \pm\sqrt{-\beta_2}(1+\beta_2)$.

Now consider $x = y = -1$, $z = 1$. The options are as follows:

* $u, v, w = \pm 1$. Again the Jacobian is diagonal, and the related eigenvalues are $-2u(vw-\beta_2), -2v(wu-\beta_2), -2w(vu+\beta_2)$, what means that for $|\beta_2| < 1$ the stability condition is HB ($uvw > 0$). For $\beta_2 > 1$ we have the configuration $u = v = -1$, $w = 1$ ('forced' HB), for $\beta_2 < -1$ we have $u = v = 1$, $w = -1$ (not HB).

* $u, v = \pm 1$ and $uv = -\beta_2$, what is not generic; the same argument for $u, w = \pm 1$ and $uw = \beta_2$, etc.

* $u = \pm 1$, $v = -w - \mp\beta_2$; then two eigenvalues are $\pm(1-\beta_2^2)$.

* $u = v = -w = \pm\sqrt{-\beta_2}$. Then, $\beta_2 < 0$ and the related eigenvalues are $e, e, -2e$, where $e = \pm(1+\beta_2)\sqrt{-\beta_2}$.

Now we are going to consider the bidirectional coupling, i.e. $\beta_1 \neq 0$, $\beta_2 \neq 0$. Yet, let us start from the special case when $x = 0$; later this option will be conveniently excluded. For any fixed point we have

$$yz + \beta_1 u = 0 \tag{10}$$
$$(1-y^2)\beta_1 v = 0 \tag{11}$$
$$(1-z^2)\beta_1 w = 0 \tag{12}$$
$$(1-u^2)vw = 0 \tag{13}$$
$$(1-v^2)(wu+\beta_2 y) = 0 \tag{14}$$
$$(1-w^2)(uv+\beta_2 z) = 0 \tag{15}$$

Then, from (11), either $v = 0$, or $y = \pm 1$. If $v = 0$, then the second column of the Jacobian reads $(z, 0, 0, 0, \beta_2, 0)$, and the fourth column $(\beta_1, 0, 0, 0, w, 0)$. As $yz + \beta_1 u = 0$ and $wu + \beta_2 y = 0$, these columns are proportional one to another, hence one of the eigenvalues is zero.

Now suppose that $v \neq 0$ and $y = \pm 1$. As $\dot{z} = (1 - z^2)\beta_1 w$, we see that either $w = 0$ or $z = \pm 1$. In the former, the third column of the Jacobian reads $(y, 0, 0, 0, 0, \beta_2)$, and the fourth one is $(\beta_1, 0, 0, 0, 0, v)$. As $yz + \beta_1 u = 0$ and $uv + \beta_2 z = 0$, we get $y/\beta_1 = \beta_2/v$, hence again one eigenvalue is zero. If, in turn, $w \neq 0$ and $z = \pm 1$, then at any fixed point we have $\dot{u} = (1 - u^2)vw = 0$. Recalling that $v \neq 0$, we get $u = \pm 1$. Then, all elements in the first column of the Jacobian are equal to zero.

Below we can assume that all weights are different from zero. In each one out of six equations (1–6) for the fixed point, one of two factors is zero. In other words, the set of equations (1–6) can be written as $\dot{x} = F(x)G(x)$ etc., where $F(x) = 1 - x^2$ and, symbolically, $G(x) = yz + \beta_1 u$. Then, all cases to be considered can be written in the form $FFGGFG$, what means: $F(x) = 0$, $F(y) = 0$, $G(z) = 0$, $G(u) = 0$, $F(v) = 0$, $G(w) = 0$. Obviously, we do not need to distinguish between $FGFGGG$ and $GFFGGG$, and so on.

*$FFFFFF$. This case is discussed in the main text. For completeness of this material recall that the Jacobian is diagonal, and once the coupling is small ($|\beta_1| < 1$ and $|\beta_2| < 1$), the condition of stability is HB for both layers: $xyz > 0$ and $uvw > 0$. Once $\beta_1 > 1$ and $|\beta_2| < 1$, the only stable solution HB (if achieved) in the layer (u, v, w) forces HB also in the layer (x, y, z), as $x = u$, $y = v$ and $z = w$ there. If $\beta_1 < -1$ and $|\beta_2| < 1$, the HB in (u, v, w) forces $x = -u$, $y = -v$ and $z = -w$ (no HB). For $\beta_1 > 1$ and $\beta_2 > 1$, the stability condition is only $x = u$, $y = v$ and $z = w$, with HB possible but accidental. If $\beta_1 > 1$ and $\beta_2 < -1$ or the opposite, we expect oscillations of the weights. This effect is more clear when the equations (1-6) are linearised around zero; we get $\dot{x} = \beta_1 u$, $\dot{u} = \beta_2 x$ etc., i.e. three mutually independent harmonic oscillators. See following section for details.

*$FFFFFG$. The Jacobian is diagonal, except the last row. The eigenvalue related to w is zero, as $uv + \beta_2 z = 0$.

*$FFFFGG$. In four first rows of the Jacobian, only diagonal elements are non-zero. The eigenvalues related to v and w are $\pm u\sqrt{(1 - v^2)(1 - w^2)}$.

*$FFGFFG$. In 1-st, 2-nd, 4-th and 5-th rows of the Jacobian, only diagonal elements are non-zero. The eigenvalues related to z and w are $\pm\sqrt{\beta_1\beta_2(1 - z^2)(1 - w^2)}$.

*$FFGGFF$. In 1-st, 2-nd, 5-th and 6-th rows of the Jacobian, only diagonal elements are non-zero. As $xy + \beta_1 w = vw + \beta_2 x = 0$, the eigenvalues related to z and u are equal to zero.

*$FFGGGF$. In 1-st, 2-nd and 6-th rows of the Jacobian, only diagonal elements are non-zero. The three eigenvalues related to z, u, v are $\pm w\sqrt{(1 - u^2)(1 - v^2)}$ and zero.

*$FFGFGG$. In 1-st, 2-nd, and 4-th rows of the Jacobian, only diagonal elements are non-zero. One eigenvalue related to z, v and w is zero; the remaining two are $\pm\sqrt{1 - w^2}\sqrt{\beta_2\beta_1(1 - z^2) + u^2(1 - v^2)}$.

*$FFFGGG$. The Jacobian has a block-diagonal structure, and the eigenvalues related to its upper left part, which is diagonal, has been discussed above. The lower right block has all diagonal elements equal to zero; the sum of its eigenvalues is zero; hence some of them are nonnegative.

*$FFGGGG$. Suppose that $x = y = +1$. As $xy + \beta_1 w = 0$, we get $w = -1/\beta_1$. Further, as $wu = -\beta_2$, we have $u = \beta_1\beta_2$; similarly $wv = -\beta_2$ gives $v = \beta_1\beta_2$. From $uv = -\beta_2 z$ we get $z = -\beta_1^2\beta_2$. As an effect, $yz + \beta_1 u = 0$, what means that the first row of the Jacobian contains only zeros. The same construction works if $x = \pm 1$ and $y = \pm 1$.

*$FGGFGG$. Solving equations (1–6) we get $xu = \beta_2\beta_1 = \pm 1$, what is not generic. Yet we can add that in the Jacobian, the second column is $(0, 0, (1 - z^2)x, 0, (1 - v^2)\beta_2, 0)$, and the sixth column is $(0, 0, (1 - z^2)\beta_1, 0, (1 - v^2)u, 0)$. Hence the 2-nd column is proportional to the 6-th one. For the same reason, the 3-rd column is proportional to the 5-th one.

*$FGGGFG$. Here $zx + \beta_1 v = 0$ and $xy + \beta_1 w = 0$, hence $yzx^2 = \beta_1^2 vw$. On the other hand

$vw = -\beta_2 x$, hence $yz = -\beta_1^2\beta_2/x$. Also $uv + \beta_2 z = 0$, then $\beta_1 u = -\beta_2\beta_1 z/v$; finally $z = -\beta_1 v/x$, then $\beta_1 u = \beta_1^2\beta_2/x$. and $yz+\beta_1 u = 0$. This means that all elements in the first row of the Jacobian are zero.

**FGGGGG*. Suppose that $x = +1$. Then $z = -\beta_1 v$, $y = -\beta_1 w$, $vw = -\beta_2$; hence $yz = -\beta_1^2\beta_2$. On the other hand, $wu = -\beta_2 y = \beta_1\beta_2 w$, hence $u = \beta_1\beta_2$. We get $yz+\beta_1 u = 0$. As other diagonal elements of the Jacobian are also zero, the sum of the eigenvalues is zero. The same reasoning works for $x = -1$.

**GGGGGG*. All the diagonal elements of the Jacobian are equal to zero. Then, its trace is zero, with the consequence as above.

We have checked that the non-generic fixed points are also unstable. To summarize, the only stable fixed points are those where all weights are ± 1.

Derivation of oscillator equation

We assume that the coupling coefficients are $\beta_1 > 1$ and $\beta_2 < -1$. It means that $\beta_1\beta_2 < -1$. Assuming, $\beta_1, |\beta_2| \gg 1$, then model equations (equation 2 in the main text) can be approximated by a system where each link is directly dependent only on its replica from the other layer:

$$\dot{x}_{ij}^{\alpha} = \Big(1 - (x_{ij}^{\alpha})^2\Big)\beta_\alpha x_{ij}^{\alpha'} \tag{16}$$

Therefore, a system comprises pairs of coupled variables. Each pair can be transformed into a one-variable derivative equation of second order:

$$\ddot{x}_{ij}^{\alpha} + \frac{(\dot{x}_{ij}^{\alpha})^2 x_{ij}^{\alpha}}{1-(x_{ij}^{\alpha})^2}\Big(2 + \frac{\beta_2}{\beta_1}\Big) - \beta_1\beta_2\Big(1 - (x_{ij}^{\alpha})^2\Big)x_{ij}^{\alpha} = 0 \tag{17}$$

Further, if $\beta_1 \approx |\beta_2|$, then (17) becomes the non-linear oscillator:

$$\ddot{x}_{ij}^{\alpha} + \frac{(\dot{x}_{ij}^{\alpha})^2 x_{ij}^{\alpha}}{1-(x_{ij}^{\alpha})^2} + \beta_1^2\Big(1 - (x_{ij}^{\alpha})^2\Big)x_{ij}^{\alpha} = 0 \tag{18}$$

On the other hand, if it is assumed that $|x_{ij}^1|, |x_{ij}^2| \ll 1$, then the pair of equations from 16 is simplified to:

$$\begin{cases} \dot{x}_{ij}^1 = \beta_1 x_{ij}^2 \\ \dot{x}_{ij}^2 = \beta_2 x_{ij}^1 \end{cases}, \tag{19}$$

which transformed to the second order equation of one variable gives equation (8) from the main text.

Supplementary Methods

Details of numerical calculations

Numerical implementation of a model presented in the main text in (1) needs one adjustment: $x_{ij}^{\alpha} \in [-1+\epsilon, 1-\epsilon]$, where $\epsilon = 10^{-5}$. The reason to introduce the parameter ϵ is to evade the situation when due to finite computation precision the derivative $\dot{x}_{ij}^{\alpha}$ becomes exactly zero at the fixed point $x_{ij}^{\alpha} = \pm 1$. Such a link would be unable to change its weight ever again regardless of the other changes in its ”neighbourhood”. With the parameter ϵ the links' weights that are very close to but not at ± 1 still can change their value.

Numerical analysis of a dynamical system requires the proper halting conditions which are directly connected to the issue of the solution stability. The solution stability can be analysed analytically as it is shown in previous subsection. Such an approach describes an expected outcome of the numerical solution, but does not give information about the time needed to reach the solution. This is a nuisance in numerical analysis and that is why the halting conditions are necessary.

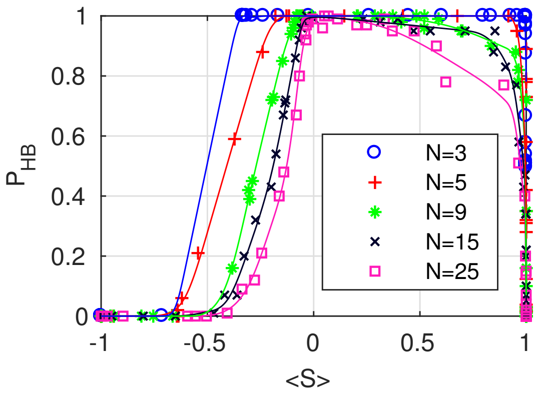

Supplementary Figure S1: Mutual relationship of the probability P_{HB} versus mean interlayer link order $\langle S \rangle$ for networks with symmetric coupling β for different network sizes N. Each data point corresponds to the same value of symmetric coupling coefficient β.

The conditions used in this paper consider two kinds of system dynamics. The system may achieve a fixed point. A solution is believed to be numerically stable when for each pair of nodes for each layer, two following inequalities are simultaneously fulfilled:

$$|x_{ij}^{\alpha}| > 0.99 \text{ and } x_{ij}^{\alpha} \cdot \dot{x}_{ij}^{\alpha} > 0. \tag{20}$$

The first condition implies that an edge weight is close to the possible stable value (± 1) and the second inequality ensures that the weight is changing towards this value. There is also a possibility that the system will never achieve such a point. Instead for example, the trajectory can be trapped around a marginally stable fixed point or even a saddle. The application of the parameter ε can make the trapping time shorter, but it is not a universal cure. Hence, applying a rather long numerical time t_{max} of dynamics is necessary.

Summing up, in this paper a single run of calculations is halted when the inequalities (20) are fulfilled or when the numerical time of the variables $[x_{ij}^{\alpha}(t)]$ exceeds the arbitrary chosen value of $t_{max} = 1\ 000$.

Heider balance probability and interlayer link order in symmetric systems

Figures 3a and 3b in the main text show the relationship of the probability P_{HB} versus mean interlayer link order $\langle S \rangle$ for networks with symmetric coupling β for different network sizes N. Presented in Supplementary Fig. S1, P_{HB} *vs* $\langle S \rangle$ provides no new information but only better perspective of data corresponding to $P_{HB}\big(\langle S \rangle (\beta)\big)$. For all network sizes the probability $P_{HB} \approx 1$ when $|\langle S \rangle| \ll 1$ but the range of high P_{HB} is not symmetrical with respect to $\langle S \rangle = 0$. This range is skew to the left and is narrower for larger N, which corresponds to a narrow peak observed in Fig. 3a for larger N. Moreover, the larger is the system the smaller is the range when $P_{HB} > 0$, and the sooner P_{HB} decays as the function of $|\langle S \rangle|$. For $N = 3$ there are such values of β that the probability $P_{HB} = 1$ and the complete ferromagnetic ILO ($\langle S \rangle = 1$) are observed simultaneously.

Relaxation time of system dynamics

Relaxation time of the whole system is the time needed to achieve a stationary or a balanced solution. It is expected to be inversely proportional to the value of the r.h.s. of (2) from the main paper; yet the latter changes in time. We have analysed relaxation time for networks networks with symmetric (i.e. $\beta_1 = \beta_2$) and antisymmetric coupling (i.e. $\beta_1 = -\beta_2$). The results are presented in Supplementary Fig. S2. This time is the longest if oscillations are possible or when the interlayer and intralayer terms in (2) from the main paper are of opposite signs and similar absolute values. Hence, in the case of systems with symmetric coupling this time increases with $|\beta|$ for $|\beta| \ll 1$, and decreases with $|\beta|$ for $|\beta| \gg 1$.

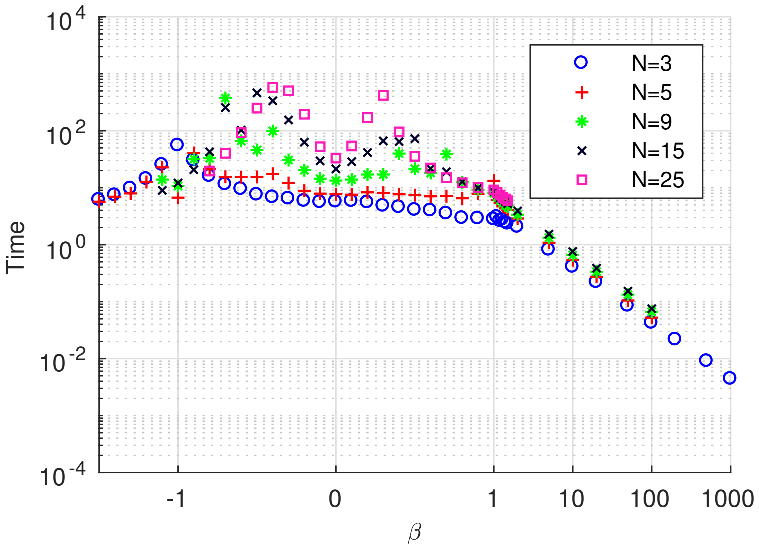

(a) $\beta \equiv \beta_1 = \beta_2$

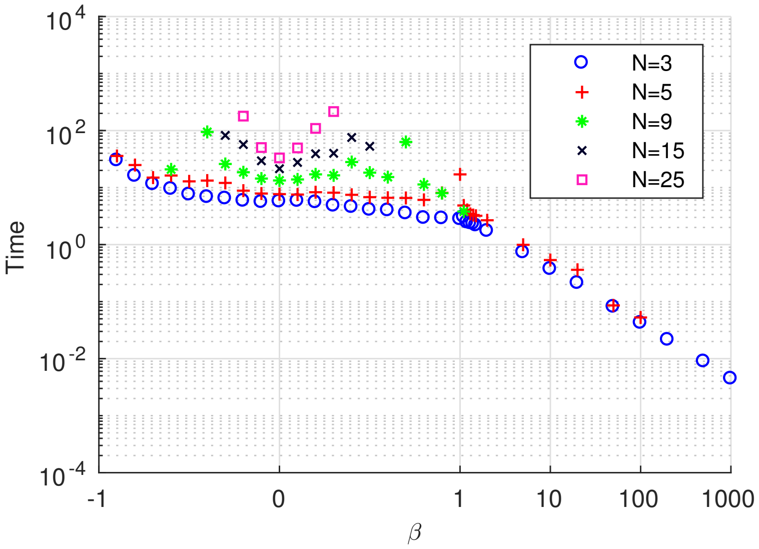

(b) $\beta \equiv \beta_1 = \beta_2$

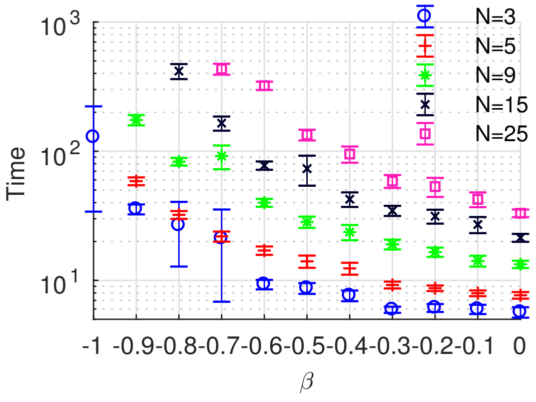

(c) $\beta \equiv \beta_1 = -\beta_2$

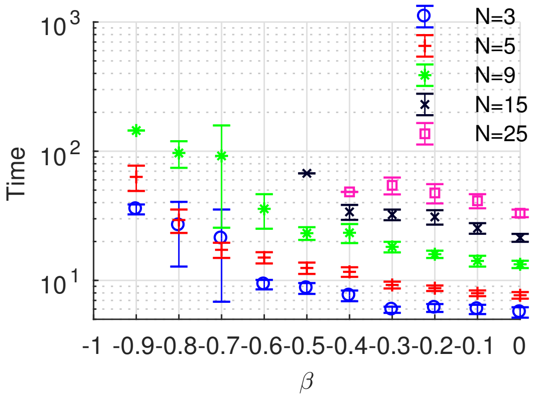

(d) $\beta \equiv \beta_1 = -\beta_2$

Supplementary Figure S2: Relaxation times for networks with symmetric (a)–(b) and antisymmetric (c)–(d) coupling. The time needed to achieve a stationary state is shown in panels (a) and (c). The time needed to achieve a balanced state is shown in panels (b) and (d). In panels (c)–(d) error bars represent standard error. In panels (a)–(b) error bars were skipped for better visibility.

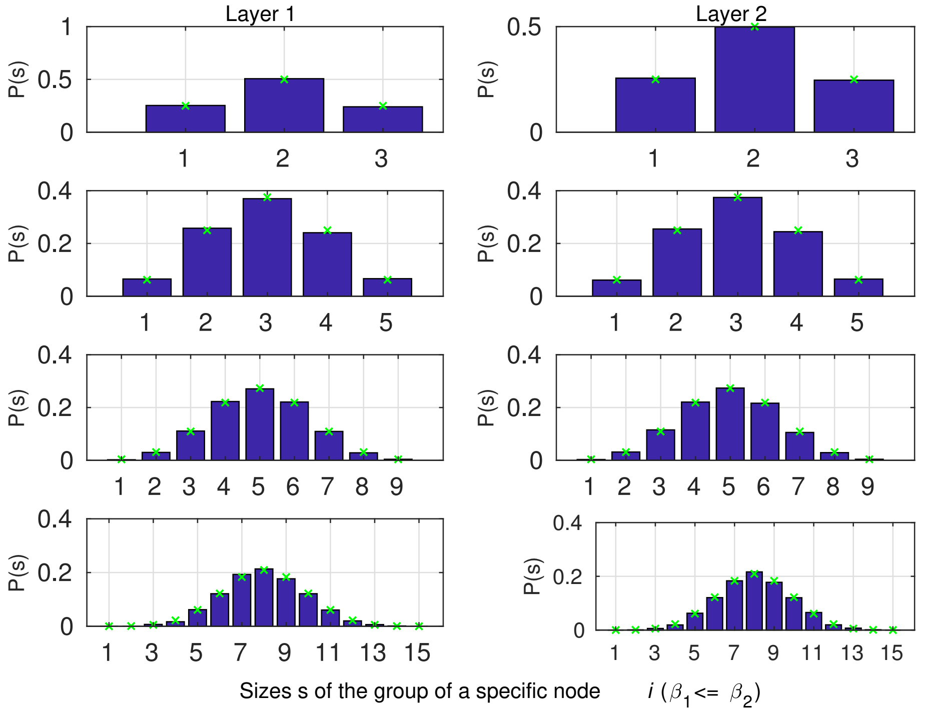

Supplementary Figure S3: Group A size distributions for different network sizes (from to top to bottom: $N = 3, 5, 9, 15$) and different layers. In each column the results are aggregated over different coupling coefficients pairs for $\beta_1 \leq \beta_2$. The blue bars represent results from 100 system realizations. x represent points given by (21).

Distribution of group sizes in balanced networks

Nodes from each layer in the HB can be divided into two groups A and B. Internally links in those groups are $+1$ and all the links connecting these groups are -1. We have analysed the distribution of group sizes for a limited number of coupling coefficient pairs $\{(-1.5, -1.5), (-1.5, -1.3), \ldots, (-1.5, 1.5), (-1.3, -1.3), \ldots, (-1.3, 1.5), \ldots, (1.3, 1.5), (1.5, 1.5)\}$ for $N = 3, 5, 9, 15$. If in any layer a stationary state with the HB was reached, we divided nodes in this layer into groups (two different divisions were possible to occur). In order to divide nodes into groups: a node $n = 1$ was always assumed to belong to the group A. We tested the hypothesis that nodes are divided into groups with equal probability. Then, the theoretical group A size distribution is related to the binomial distribution:

$$P(|A| = s) = \Big(B(N, s) + B(N, N - s)\Big)\frac{s}{N}, \qquad (21)$$

where $B(N, s)$ is a binomial distribution of having s successes in N trials with the probability 0.5.

The above way of dividing nodes skews the group A size distribution towards larger sizes, because the chosen node is more probable to belong to the larger group. Moreover, group A can't be empty, but it is possible to have all the nodes belonging to A (paradise). Using Pearson's chi-square test we estimated p-values for each N, for each layer and for each measured coupling coefficient pair when the stationary state with the HB is achieved for at least 20% of the system runs. Afterwards, using Holm-Bonferroni[1] correction with significance level of 0.05 only in one case ($N = 3, \beta_1 = -1.5, \beta_2 = -1.1, \alpha = 1$) the hypothesis should be rejected.

We also grouped all the results over coupling coefficient pairs for different N and different layers (Supplementary Fig. S3). The differences between the expected and real distributions are small and using Holm-Bonferroni correction with significance level of 0.05 the hypothesis that group A size distribution is given by equation (21) could not be rejected (Supplementary Table 1). Thus, the process of dividing nodes into groups is random and does not depend on the system parameters.
(1) Holm, S. (1979). "A simple sequentially rejective multiple test procedure". Scandinavian Journal of Statistics. 6 (2): 65–70.

Supplementary Table 1: Testing conformance of observed distribution of group A sizes for different number of nodes N and layer α with expected distribution given by (21). The values in the table represent p-values of Pearson's chi-square test.

N	α	
	1	2
3	0.099	0.26
5	0.11	0.43
9	0.76	0.60
15	0.56	0.59
